# Factor structure of the Parent Emotional Reaction Questionnaire: analysis and validation

**DOI:** 10.3402/ejpt.v6.28733

**Published:** 2015-09-01

**Authors:** Tonje Holt, Judith A. Cohen, Anthony Mannarino

**Affiliations:** 1 Norwegian Centre for Violence and Traumatic Stress Studies, Oslo, Norway; 2 Center for Traumatic Stress in Children and Adolescents, Allegheny General Hospital, Pittsburgh, PA, USA

**Keywords:** Parents, emotional reactions, children, trauma, factor analysis

## Abstract

**Background:**

Although many children experience violence and abuse each year, there is a lack of instruments measuring parents’ emotional reactions to these events. One instrument, the Parent Emotional Reaction Questionnaire (PERQ), allows researchers and clinicians to survey a broad spectrum of parents’ feelings directly related to their children's traumatic experiences. The objectives of this study were: (1) to examine the factor structure and the internal consistency of the PERQ; (2) to evaluate the discriminant validity of the instrument; and (3) to measure whether potential subscales are sensitive to change.

**Method:**

A Norwegian sample of 120 primary caregivers of a clinical sample of 120 traumatized children and youths (*M* age=14.7, SD=2.2; 79.8% girls) were asked to report their emotional reactions to their child's self-reported worst trauma. Exploratory factor analysis was used to explore the underlying factor structure of the data.

**Results:**

The analysis of the PERQ showed a three-factor structure, conceptualized as PERQdistress, PERQshame, and PERQguilt. The internal consistencies of all three subscales were satisfactory. The correlations between the PERQ subscales and two other parental measurements revealed small to moderate effect sizes, supporting the discriminant validity of the PERQ subscales. The differences in sum scores of the PERQ subscales before and after a therapeutic intervention suggest that all of the subscales were sensitive to change.

**Conclusions:**

Study findings support the validity of conceptualizing the PERQ as three separate subscales that capture clinically meaningful features of parents’ feelings after their children have experienced trauma. However, the subscales need to be further evaluated using a larger sample size and a confirmatory factor analytic approach.

A significant number of children and adolescents exposed to potentially traumatic events, such as sexual abuse, domestic violence, and peer violence, develop mental health problems (Dube, Felitti, Dong, Giles, & Anda, 2003; Fairbank & Fairbank, 2009; McLaughlin et al., 2013). Studies have shown that parental functioning and stress reactions influence the occurrence and persistence of children's posttrauma symptoms (Davis & Siegel, 2000; Dyb, Jensen, & Nygaard, 2011; Laor, Wolmer, & Cohen, 2001; Salmon & Bryant, 2002; Scheeringa & Zeanah, 2001; Trickey, Siddaway, Meiser-Stedman, Serpell, & Field, 2012). However, these studies have primarily investigated children's reactions to parents’ posttrauma or mental health symptoms. Studies focusing on caregivers’ diverse emotional reactions to the experiences of their children are few. To facilitate new research in the area of child and adolescent trauma, the field needs validated instruments to assess parents’ emotional reactions to trauma.

Studies show that when children are exposed to traumatic incidents, their parents may report and experience psychological stress (Cabizuca, Marques-Portella, Mendlowicz, Coutinho, & Figueira, 2009; Davies, 1995; Elliot & Carnes, 2001; Kelley, 1990; Newberger, Gary, Waternaux, & Newberger, 1999). Most commonly, studies about parental stress focus on parents’ posttraumatic stress (PTS) symptoms. However, it is probable that parents are overwhelmed by a broad spectrum of emotional reactions when their children have experienced violence, abuse, and other types of traumatizing events. They may feel distress and guilt because they did not prevent the terrifying or unacceptable event from happening. Parents may also experience inadequacy and discomfort if their children develop problems and behaviours that challenge their normal parenting skills. Parents who have previously been exposed to abuse and/or violence may also experience a reactivation of the feelings associated with their own traumas. Furthermore, parents may experience shame and fear that their children will recount their traumatic experiences to persons outside the family, and some may feel anger, for example, at the perpetrator for harming the child. These various parental reactions may be understood in light of theories that emphasize that the primary role of caregivers is to protect their children (Bowlby, 1982; Pynoos, Steinberg, & Piacentini, 1999). For example, Pynoos et al. (1999) emphasize that parents have a role as protective shields for their children. When trauma affects children, it is thus understandable that the parents may feel that they have failed in their primary role as protectors. This failure may lead to guilt, frustration, and sadness. When the parents additionally feel that they cannot help and/or comfort their children in the aftermath of trauma, they may experience elevated levels of stress.

Emotions are distinguished from each other by characteristics such as physiology, automatic appraisal, developmental appearance, onset, occurrence, duration, distinctive thoughts, and subjective experiences (Ekman, 1999; Tomkins, 1962). Lewis (2008) differentiates between basic emotions—such as sadness and anger—and self-referring emotions—such as guilt and shame. It is reasonable to assume that parents’ emotional reactions related to their children's trauma exposure are as diverse and complex as other emotional experiences. Parents may exhibit not only basic emotions but also self-referring emotions, because their children's trauma may reflect on their role as parents. To our knowledge, only one instrument has the ability to measure a diverse spectrum of caregivers’ emotional responses to their children's traumatic experiences. This instrument, the Parent Emotional Reaction Questionnaire (PERQ; Mannarino & Cohen, 1996), was originally designed to measure parental reactions in relation to sexual abuse, but the instrument's wording was later revised to encompass more general traumatic experiences. The instrument has previously been used in treatment studies (Cohen, Deblinger, Mannarino, & Steer, 2004; Cohen & Mannarino, 1996, 2000; Deblinger, Mannarino, Cohen, & Steer, 2006), and findings from these studies have shown that parents’ emotional reactions are related to their children's clinical outcomes. The scale has also been shown to be sensitive to change and useful in effectiveness studies conducted in ordinary community clinics (Holt, Jensen, & Wentzel-Larsen, 2014).

Although the PERQ consists of items that describe different types of emotional reactions, the scale was developed and has only been used as one unified scale composed of one factor. To date, no published study has evaluated the factor structure of the instrument. Neither has any study examined the discriminant validity of the measure nor the extent to which the measure is not unduly related to other similar, yet distinct, constructs. Furthermore, the instrument has not been applied in a Norwegian context or in samples of children who have experienced a wide range of traumatic experiences. Thus, in the current study, the underlying factor structure, the internal consistency, the discriminant validity, and the change sensitivity of the Norwegian translated version of the PERQ were evaluated in a sample of 120 caregivers of children and adolescents who had been exposed to various traumatic experiences.

The present study is exploratory in nature. Still, based on the assumption that the emotional reactions conceptualized in the PERQ are as diverse and complex as other emotional experiences, there is reason to believe that the PERQ items will constitute more than one scale. It is also reasonable to conjecture that the emotional reactions of parents measured by potential PERQ subscales capture distinct constructs that differ from what other parental measurements capture and that these potential subscales are sensitive to change.

## Aims of the present study

The overarching goals of the current study are to evaluate the Parent Emotional Reaction Questionnaire (PERQ) and to suggest improvements to further develop the scale. We sought to learn more about the underlying factor structure of the PERQ, which in turn would give insight into the underlying structure of the emotions experienced by parents of children exposed to trauma. First, we investigated the factor structure of the PERQ by using exploratory factor analysis (EFA) and examining the internal consistency of potential subscales. Second, to determine the discriminant validity of the PERQ, the scale's association with parents’ depressive symptoms, (measured using the Center for Epidemiologic Studies Depression Scale, CES-D: Radloff, 1977) and self-reported parental support (measured using the Parental Support Questionnaire, PSQ; Mannarino & Cohen, 1996) was investigated. Third, we examined whether potential subscales were sensitive to change by measuring the change in the sum scores of the potential subscales before and after a therapeutic intervention. The following three research questions were therefore developed:

1. Do the items on the PERQ constitute more than one subscale?
2. What is the relationship between the PERQ subscales and parents’ depressive symptoms and parental support?
3. Are potential subscales of the PERQ sensitive to change?

## Method

### Participants

The sample was part of a larger effectiveness study that had a primary objective to study the short- and long-term effects of Trauma-Focused Cognitive Behavioral Therapy (TF-CBT) for traumatized children and adolescents between 10 and 18 years of age. In total 146 caregivers were asked to complete the PERQ questionnaire at the beginning of therapy (T1) and approximately 6 months after T1 (post-therapy; T2). In 16 cases, the caregiver did not return or answer the questionnaire. Ten additional cases were excluded from the analyses, because the questions were answered by caregivers who were either perpetrators of the trauma or foster parents who did not know the child when the trauma occurred. Therefore, the PERQ was completed at T1 by 120 eligible parents or close familial caregivers of 120 children and adolescents. At T2, 89 of the same parents or caregivers completed the questionnaire.

A detailed description of the sample is presented in Table 1. The majority of the caregivers were mothers (79.2%), 18.3% were fathers, and 2.5% were other caregivers (i.e., an older sibling, grandmother, or stepmother) of the traumatized child. In addition, most of the caregivers were Norwegian (82.5%). The youth ranged in age from 10 to 18 years (*M*=14.7, SD=2.2), and 80% were girls. More than half of the youth (55.8%) lived in a single-parent household with their mother. On average, the youth reported having been exposed to 3.4 (SD=1.8, range 1–8) different types of traumatic events. When asked to rank their “worst trauma,” the largest group of youth (28.3%) reported intrafamilial violence. On average, the time since the “worst” trauma had occurred was 30 months (SD: 33, range: 1–138). Although the youth had experienced different types of trauma, they all presented significant levels of PTSD symptoms (scores of 15 or above on the Child PTSD Symptom Scale, CPSS; Foa, Johnson, Feeny, & Treadwell, 2001) and reported experiencing at least one symptom of each of the three PTSD symptom criteria: reexperiencing, avoidance, and hyperarousal.

**Table 1:** Descriptive statistics for participating caregivers and children

Demographics of the children (*N*=120)	*n* (%)
Person who completed the questionnaire (*n*=120)	
Mother	95 (79.2)
Father	22 (18.3)
Others	3 (2.5)
Caregivers’ employment situation (*n*=113; lower *n* due to missing data)	
Working full-time	59 (52.2)
Working part-time	18 (15.9)
Job seeker	4 (3.5)
Student	5 (4.4)
Welfare recipient/others	27 (23.9)
Caregivers’ educational level (*n=*114; lower *n* due to missing data)	
Completed junior high school	17 (14.9)
Completed high school	41 (36)
Completed vocational school	15 (13.2)
≤4 years of college/university	36 (31.6)
>4 years of college/university	5 (4.4)
Families’ contact with child welfare system (*n=*112; lower *n* due to missing data)	
Contact with child welfare system last 2 months	32 (28.6)
No contact with child welfare system last 2 months	80 (71.4)
Caregivers’ ethnicity (*n*=120)	
Norwegian	99 (82.5)
Asian	9 (7.5)
Western European	3 (2.5)
Eastern European	2 (1.7)
African	3 (2.5)
South/Central American	3 (2.5)
Northern American	1 (0.8)
Demographics of the children (*N*=120)	*n* (%)
Children's gender (*n*=120)	
Girls	96 (80)
Boys	24 (20)
Children's age (*n*=120)	
Range	10–18
Mean, SD	*M*=14.7, SD=2.2
Children's living situation (*n*=120)	
Lives with both parents	31 (25.8)
Alternates between living with mother and father	4 (3.3)
Lives mostly or only with mother	67 (55.8)
Lives mostly or only with father	13 (10.8)
Foster care	2 (1.7)
Others (alone, institution, with boyfriend or girlfriend)	3 (2.5)
Children's primary (worst) trauma (*n*=120)	
Accident	3 (2.5)
Sudden death/injury of a close person	21 (17.5)
Hospitalization	1 (0.8)
Peer violence	22 (18.3)
Robbery	1 (0.8)
War/refugee	1 (0.8)
Witnessed intra-familial violence	4 (3.3)
Exposed to intra-familial violence	30 (25)
Intra-familial sexual abuse	11 (9.2)
Extra-familial sexual abuse	26 (21.7)
Time since primary trauma occurred, in months (*n*=120)	
Range	1–138
Mean, SD	*M*=30 months, SD=33
Children's total number of traumatic experiences (*n*=120)	
Range	1–8
Mean, SD	*M*=3.4, SD=1.8
Children's scores on the Child PTSD Symptom Scale (CPSS)	
Range	15–46
Mean, SD	*M*=37.4, SD=7.6

Traumas inside the family (intra-familial) are defined as occurrences in which the perpetrator was a caregiver of the child and lived with him or her (in this study, the intra-familial perpetrator was the father, mother or step-father of the child).

### Procedure

The study was approved by the Regional Committee for Medical and Health Research Ethics. Written, active consent to participate was given by both the children and their caregiver. Normal referral procedures were followed, and all of the children were referred for treatment by their primary physician or by Child Protective Services. To be eligible for the study, the youth had to have experienced at least one potentially traumatizing event and be suffering from significant PTS reactions. Exclusion criteria were acute psychosis, active suicidal behaviour, documented intellectual disability, or the need for an interpreter. To assess traumatic experiences, we developed a checklist based on the items described in the Traumatic Events Screening Inventory for Children (TESI-C; Ribbe, 1996). Many of the children had experienced more than one traumatic event and were, therefore, asked to identify what they considered to be their worst trauma or the trauma that disturbed them the most.

The parent who accompanied the child or adolescent to the first treatment session at the child guidance clinic completed the PERQ at T1. The PERQ was again completed by the same parent after the child had completed 15 treatment sessions (T2). Parents were instructed to answer the questionnaire in response to the child's worst trauma, which was identified during the TESI interview. Most of the parents answered the questions on a computer. A clinical psychologist offered to assist them if they needed help or had any questions during the process of completing the instrument. If the parent was not present at the treatment session, a paper version of the questionnaire was either given to the child or mailed to the caregiver to be returned in a stamped envelope provided with the questionnaire. If the questionnaire was not returned the following week, a research assistant attempted to conduct the assessment over the phone.

### Measures

#### Parent Emotional Reaction Questionnaire

The PERQ measures a set of parental emotional reactions related to his/her child's worst traumatic experience. The caregivers were asked to rate their feelings on a 5-point Likert scale ranging from 1 to 5 (1=never, 5=always), indicating how often they experienced each emotional reaction during the last 2 weeks. Higher scores indicate more frequent emotional reactions to the child's worst traumatic experience. The scale's authors previously found the PERQ to have good validity and reliability (Mannarino & Cohen, 1996). The scale's internal consistency was *α*=0.87, and its test–retest reliability was *r*=0.90 (Mannarino & Cohen, 1996).

The English language version of the PERQ was translated into Norwegian partly following the translation methodology described by Hilton and Skrutkowski (2002). First, two project members who are specialists in the field of child and parental trauma reactions translated the instrument from English into Norwegian. Second, the instrument was modified to better suit the structure of the Norwegian language. Third, the scale was translated back into English by someone who did not take part in the initial translation. Finally, the back-translated version of the scale was evaluated to ensure that the conceptual meaning of each item had been maintained. The english version of the scale and the Norwegian translation of the scale are presented in the appendix.

The translated version of the instrument was based on the revised version of the instrument, meaning that the wording of the items captured parental emotional reactions, related not only to child sexual abuse but to all traumatic experiences. The revised instrument consists of 15 items. However, the last item in the scale, “I feel guilty that I did not know about the trauma sooner,” was excluded from the analyses because it was left unanswered by more than half of the participants, probably because many of the caregivers in the current sample had learned about the trauma immediately after it occurred. As such, the item was not relevant to many of the parents in this sample. A scale score was computed as the mean of the remaining 14 items.

#### Center for Epidemiologic Studies Depression Scale

Caregivers’ symptoms of depression were measured using the CES-D. The scale was developed in 1977 (Radloff, 1977) and consists of 20 self-reported items designed to measure depressive symptoms in the general adult population. The Norwegian version was developed by Clausen and Slagsvold (2005). The caregiver was instructed to indicate how often he/she felt or behaved in a given way during the last week on a 4-point Likert scale ranging from 0 to 3 (0=rarely or none of the time/less than 1 day, 3=most or all of the time/5–7 days). Although not constituting a clinical diagnosis of depression, scores at or above 16 on the CES-D are considered to be indicative of clinically significant symptoms of depression. The scale has previously been shown to have good internal consistency (*α*=0.85 for the general population and *α*=0.90 for the clinical population; Radloff, 1977). The current study yielded an internal consistency score of *α*=0.92.

#### Parental Support Questionnaire

The PSQ (Mannarino & Cohen, 1996) is a self-reported evaluation of the support parents give to their children after a traumatic experience. The instrument measures parents’ perception of their own supportive behaviour towards their children (PSQsupport; eight questions) and assesses whether the parent blames the perpetrator or child for the trauma (PSQblame; nine questions). As with the PERQ, caregivers were asked to rate their responses to each question on a 5-point Likert scale ranging from 1 (never) to 5 (always) to indicate how often they provided support to their children during the last 2 weeks. The research group translated and back translated the instrument into Norwegian in collaboration with the scale's developers. Of the 19 items in the original instrument, 17 items were used in the Norwegian version. In the current study, the internal reliability was *α*=0.84 for the PSQ support and *α*=0.67 for the PSQ blame scale.

### Data analyses

Descriptive data included investigation of skewness, kurtosis, frequencies, mean, and correlations. Descriptive analyses were conducted on both the data gathered at T1 and T2. To explore the underlying factor structure of the data, an EFA consisting of principal axis factoring (PAF) with oblimin rotation was conducted on the PERQ's 14 items at T1 and T2. Subsequently, the same procedure was conducted only for the mothers in the sample at T1. PAF is classified as an EFA, where the goal is to model only the shared variance in a set of X measurements (Tabachnick & Fidell, 2007). The method of oblimin rotation was selected because it allows the subscales to be correlated. Sum scores of each of the revealed factors were calculated based on the raw scores. Relationships between the sum scores of each subscale and sum scores of the other parental variables were calculated using Pearson product moment coefficients (*r*). No clear cut-off values exist for determining discriminant validity, but the guidelines of Cohen (1988) suggest that *r*=0.10, 0.30, and 0.50 represent small, medium, and large effect sizes (ES), respectively. Paired-sample *t*-tests were conducted to evaluate the subscales’ sensitivity to change from pretreatment (T1) to posttreatment (T2). Only data from participants who had completed both questionnaires were analyzed. Between T1 and T2, the participants received treatment (either TF-CBT or therapies usually provided in Norwegian child and adolescent clinics). The magnitude of change in the potential PERQ subscales was measured using Cohen's *d* (*d*). Cohen (1988) suggests an ES of around *d*=0.2 to be a small ES, around *d*=0.5 to be a medium ES, and around *d*=0.8 to be a large ES. The descriptive analysis, the EFA, the correlation analysis, and the paired-sample *t*-tests were performed using SPSS version 17 (IBM SPSS Statistics, 2011).

## Results

The inter-item Pearson correlations, mean, standard deviations, and factor loadings for the 14 items constituting the PERQ were calculated. Seven of the item means were above the mean of the scale score at T1, and seven were above the scale score mean at T2. The items displayed inter-item correlations between *r*=0.02 and *r*=0.74 at T1 and between *r*=0.11 and *r*=0.75 at T2. Almost all the items displayed significant inter-item correlation coefficients at *p*<0.01 level. The exception was item 10, “I have felt embarrassed about my child's traumatic experience,” and item 12 “I have felt ashamed about my child's traumatic experience,” which yield only five and six significant inter-item correlation coefficients at *p*<0.01 level at T1 (Table 2). The investigation of the skewness, kurtosis, frequencies, mean, and inter-item correlations showed that none of the items displayed considerable violations to univariate normality at T1 or T2 (skewness<3, kurtosis<10; Kline, 2010).

**Table 2:** Inter-item correlations between the 14 PERQ items at T1 (below the diagonal) and inter-item correlations between the 14 PERQ items at T2 (above the diagonal)

Perq-Items	1	2	3	4	5	6	7	8	9	10	11	12	13	14
1. Upset	–	0.69	0.75	0.29	0.45	0.45	0.40	0.71	0.38	0.09	0.47	0.14	0.37	0.41
2. Work	0.54	–	0.60	0.38	0.42	0.41	0.48	0.57	0.46	0.27	0.58	0.33	0.47	0.48
3. Sad	0.55	0.56	–	0.37	0.50	0.48	0.50	0.61	0.38	0.20	0.55	0.27	0.33	0.37
4. Others think	0.21	0.29	0.35	–	0.43	0.48	0.55	0.44	0.44	0.38	0.57	0.62	0.41	0.52
5. Did not stop	0.32	0.33	0.34	0.35	–	0.51	0.50	0.44	0.49	0.40	0.40	0.47	0.71	0.47
6. Afraid	0.39	0.32	0.40	0.42	0.50	–	0.68	0.54	0.43	0.37	0.44	0.53	0.41	0.70
7. Sleep	0.49	0.55	0.52	0.26	0.38	0.35	–	0.50	0.67	0.37	0.47	0.50	0.43	0.63
8. Angry	0.33	0.31	0.33	0.28	0.42	0.33	0.37	–	0.43	0.11	0.53	0.23	0.35	0.54
9. Headache, etc.	0.54	0.53	0.42	0.23	0.23	0.36	0.67	0.33	–	0.27	0.58	0.40	0.33	0.50
10. Embarrassed	0.07	0.12	0.23	0.59	0.31	0.19	0.10	0.02	0.06	–	0.17	0.80	0.32	0.41
11. Cried	0.41	0.31	0.51	0.30	0.22	0.28	0.42	0.29	0.39	0.25	–	0.41	0.23	0.48
12. Shame	0.12	0.21	0.28	0.48	0.28	0.24	0.17	0.07	0.18	0.74	0.31	–	0.37	0.56
13. Responsible	0.28	0.12	0.30	0.35	0.64	0.37	0.20	0.15	0.06	0.32	0.22	0.41	–	0.38
14. Insecure	0.39	0.29	0.38	0.34	0.28	0.41	0.44	0.24	0.37	0.10	0.32	0.24	0.32	–

T1; Correlations >0.23 have *p*<0.01, T2; Correlations >0.29 have *p*<0.01.

### Exploratory factor analysis

The suitability of the data for factor analysis was assessed prior to the analysis. The Kaiser–Meyer–Oklin was above the recommended value of 0.60 at both T1 and T2 (Kaiser, 1974). Furthermore, Barlett's test of sphericity (Barlett, 1954) reached statistical significance at both time points, supporting the factorability of the correlation matrix. Furthermore, at both time points, the EFA revealed the presence of three factors with eigenvalues exceeding Kaiser's (1974) criteria of one. Together these factors explained 61.1% of the variance (38.0, 14.6, and 8.5%, respectively) at T1 and 69.4% (49.3, 12.8, and 7.3%, respectively) at T2. Inspection of the scree plot showed an elbow break after the third factor at both time points. The sample was shown to be sufficiently large (*n*=120 and *n*=89), containing more than five participants per item (Costello & Osborne, 2005). Table 3 shows the factor structure of the 14 PERQ items tested at both time points. At T1, the first factor, labelled as PERQdistress, consisted of eight items. The second factor, PERQshame, consisted of three items. The last three items constituted the PERQguilt factor. These three factors showed satisfactory internal consistency: *α*=0.85 for PERQdistress, *α*=0.81 for PERQshame, and *α*=0.75 for PERQguilt at T1. At T2 the fear item (item 6) was more equivocal than at T1, and the anger-item had higher loadings onto the distress factor than at T1. The PERQdistress subscale displayed the highest average score (*M*=3.05 at T1), and the subscale of PERQshame displayed the lowest average score (*M*=1.67 at T1). The results were comparable when only including the mothers in the T1-analysis; three factors revealed, explaining 60% of the variance (36.1, 14.7, and 9.1%, respectively).

**Table 3:** Pattern and structure matrix for EFA with oblimin rotation of a three-factor solution of PERQ items at T1 and T2

Items, T2	Pattern coefficients			Structure coefficients			Comm.
	
	Fact. 1	Fact. 2	Fact. 3	Fact. 1	Fact. 2	Fact. 3	
9. Headache, etc.	**0.84**	−0.02	0.17	**0.75**	0.12	−0.21	0.59
7. Sleep	**0.78**	−0.05	−0.01	**0.78**	0.15	−0.35	0.61
2. Work	**0.72**	0.03	0.05	**0.70**	0.19	−0.29	0.49
1. Upset	**0.69**	−0.09	−0.10	**0.72**	0.12	−0.38	0.52
3. Sad	**0.65**	0.13	−0.06	**0.71**	0.31	−**0.40**	0.53
11. Cried	**0.52**	0.21	0.03	**0.56**	0.33	−0.28	0.35
14. Insecure	**0.43**	0.05	−0.19	**0.52**	0.22	−**0.40**	0.31
8. Angry	0.39	−0.11	−0.25	**0.47**	0.08	−0.39	0.27
10. Embarrassed	−0.08	**0.96**	0.02	0.15	**0.94**	−0.30	0.89
12. Shame	0.05	**0.77**	−0.04	0.26	**0.80**	−0.35	0.65
4. Others think	0.19	**0.50**	−0.19	0.39	**0.62**	−**0.45**	0.47
5. Did not stop	0.06	0.00	−**0.81**	**0.43**	0.31	−**0.84**	0.71
13. Responsible	−0.11	0.17	−**0.73**	0.26	0.41	−**0.74**	0.57
6. Afraid	0.32	0.04	−**0.41**	**0.52**	0.27	−**0.57**	0.41
Eigenvalues	5.32	2.04	1.20				
% of variance	38.00	14.56	8.54				
Items, T1	Pattern coefficients			Structure coefficients			Comm.
	
	Fact. 1	Fact. 2	Fact. 3	Fact. 1	Fact. 2	Fact. 3	

9. Headache, etc.	**0.54**	0.26	0.00	**0.63**	**0.44**	0.35	0.45
7. Sleep	**0.54**	0.37	0.06	**0.69**	**0.56**	**0.44**	0.61
2. Work	**0.66**	−0.04	0.18	**0.74**	0.23	**0.50**	0.57
1. Upset	**0.82**	−0.35	0.21	**0.81**	−0.02	**0.51**	0.77
3. Sad	**0.74**	−0.14	0.15	**0.76**	0.15	**0.47**	0.61
11. Cried	**0.76**	0.17	−0.17	**0.73**	0.37	0.25	0.57
14. Insecure	**0.50**	0.41	0.04	**0.66**	0.59	0.41	0.59
8. Angry	**0.81**	−0.11	0.05	**0.80**	0.17	0.42	0.65
10. Embarrassed	−0.10	**0.67**	0.24	0.24	**0.70**	0.39	0.54
12. Shame	0.05	**0.88**	0.12	0.40	**0.93**	**0.41**	0.89
4. Others think	0.40	**0.45**	0.03	**0.57**	**0.59**	0.36	0.51
5. Did not stop	0.19	0.16	**0.65**	**0.56**	0.42	**0.79**	0.69
13. Responsible	0.01	0.09	**0.80**	**0.43**	0.33	**0.83**	0.70
6. Afraid	**0.49**	0.34	0.12	**0.66**	0.54	**0.46**	0.56
Eigenvalues	6.90	1.79	1.03				
% of variance	49.28	12.80	7.33				

*N*=120. Factor loadings>0.40 are in boldface. Fact.=Factors. Comm.=Communalities.

### Discriminant validity

The discriminant validity of the PERQ scales was measured by correlating the sum scores of the PERQ subscales with the sum scores of two other parental measures gathered at T1. Table 4 shows the correlations among the three PERQ subscales, the parental depression (CES-D) and parental support (PSQ-S and PSQ-B) instruments. Although they shared some common variance, the coefficients showed that these constructs are not highly correlated. Overall, the results provide support for the discriminant validity of the PERQ subscales. The correlation coefficients revealed small and medium ES (correlations between *r*=−0.16 and *r*=0.41). However, one exception emerged: the correlation coefficient between the PERQdistress and CES-D was *r*=0.51.

**Table 4:** Pearson correlation coefficients between the three PERQ subscales and the parental depression and parental support measures

	Mean	SD	PERQD	PERQG	PERQS	CES-D	PSQ-B	PSQ-S
PERQD	24.36	6.41	–					
PERQG	7.83	3.35	0.50 (120)	–				
PERQS	5.00	2.82	0.34 (120)	0.46 (120)	–			
CES-D	18.10	11.15	0.51 (115)	0.33 (115)	0.29 (115)	–		
PSQ-B	35.45	5.73	0.27 (117)	0.18 (117)	−0.16 (117)	0.10 (115)	–	
PSQ-S	35.15	4.61	0.41 (118)	0.20 (118)	0.22 (118)	0.03 (115)	0.09 (117)	–

PERQD=PERQdistress; PERQS=PERQshame; PERQG=PERQguilt; CES-D=Center for Epidemiologic Studies Depression Scale; PSQ-B=Parental Support Questionnaire, blame subscale; PSQ-S=Parental Support Questionnaire, support subscale. Correlations ≥0.29 have *p*<0.01.

### Sensitivity to change from pre- to post-therapy

There was a statistically significant change in all PERQ subscales when participants had received therapy. The change was measured from pre- (T1) to post-therapy (T2). For the PERQdistress subscale, the T1 scores (*M*=23.94, SD=6.75) were significantly different from the T2 scores (*M*=19.07, SD=7.22); *t*(86)=6.20, *p*<0.000. The T1 PERQ-shame scores (*M*=4.76, SD=2.57) were also significantly different from the PERQshame T2 scores (*M*=4.16, SD=1.98); *t*(86)= 2.65, *p*=0.010. Finally, the PERQguilt scores at T1 (*M*=8.13, SD=3.44) were significantly different from the PERQguilt scores at T2 (*M*=6.67, SD=3.11); *t*(86)=4.41, *p*=0.000. The change analyses are presented in Table 5.

**Table 5:** Descriptive statistics and the *t*-tests results for the T1 and T2 differences of PERQdistress, PERQshame, and PERQguilt

	Pre-test		Post-test		95% CI for mean difference	*t*	df	*d*
	
	*M*	SD	*M*	SD				
Distress	23.94	6.75	19.07	7.22	3.31, 6.44	6.20***	86	0.66
Shame	4.76	2.57	4.16	1.98	0.15, 1.05	4.41*	86	0.28
Guilt	8.13	3.44	6.67	3.11	0.80, 2.12	2.65***	86	0.47

* *p*<0.05

** *p*<0.001.

*d*=calculated based on the differences between T1 and T2: $\frac{\text{T}1-\text{T}3}{SD}$ where SD is the standard deviation of differences.

## Discussion

In the present study, an EFA revealed that the 14-item PERQ comprises three distinct factors, all showing satisfactory internal consistency. These subscales were conceptualized as “PERQdistress,” “PERQshame,” and “PERQguilt.” Furthermore, correlational analyses confirmed that the clustering of the three PERQ scales had satisfactory discriminant validity, and change analyses showed that all three subscales were sensitive to change.

The appearance of three factors in the PERQ confirmed our hypothesis that the PERQ items clustered along some definable emotions. This finding is in agreement with Ekman's (1999), Tomkins’ (1962), and Lewis’ (2008) theories, which propose that emotions are distinguishable on many levels. The PERQdistress, PERQshame, and PERQguilt subscales represent distinct aspects of subjective experiences, appraisals, and the self-reflective functions resulting from them. The PERQdistress items revealed the highest average mean score among the factors, indicating that distress is the most salient emotion felt by parents after their children have experienced trauma. The PERQdistress subscale includes questions about sadness, concentration difficulties, crying, feeling upset, rumination, and somatic symptoms, such as headaches and sleeping difficulties. These items are similar to the symptoms of depression described in the Diagnostic and Statistical Manual of Mental Disorders (DSM-IV; American Psychiatric Association, 2000), although depressive symptoms are not as directly linked to a child's trauma as the PERQ items are. In contrast to the PERQshame and PERQguilt items, the items in the PERQdistress measure a construct that may lead to a loss of interest and pleasure in daily activities. Furthermore, in line with Lewis’ distinction between self-referring and non-self-referring emotions (Lewis, 2008), the PERQdistress items are not assessing emotions that reflect back on the self. However, it is worth mentioning that the item assessing parents’ feelings of anger had relatively weak loadings on all of the factors, although the loading onto the distress factor was highest.

All PERQshame items embody a sense of being ashamed or embarrassed, and this factor appears to be the most clearly defined of the three factors. The items differ from the PERQdistress and PERQguilt items primarily because they require that another individual observe the child's trauma (a public audience). The division of the items into the PERQshame and PERQguilt subscales is reasonable considering the theoretical perspectives that distinguish between these emotions. For instance, Tracy and Robins (2004) propose that shame includes a “negative evaluation of the self,” whereas guilt embodies a “negative evaluation of a specific behaviour.” Empirical research supports this distinction (Tangney, Stuewig, & Mashek, 2007). Tangney et al. (2007) indicate that appraisals of “*I* did that horrible thing” (shame) instead of “I *did* that horrible *thing*” (guilt) often result in different subjective experiences, motivations, and behaviours. It is also proposed that shame appears to be less adaptive than guilt because shame often results in attempts to deny, hide, or escape, whereas guilt often results in efforts to confess, apologize, and attempt to undo or repair the consequences of a behaviour (Baumeister, Stillwell, & Heatherton, 1994; Flicker & Barlow, 1996; Ketelaar & Au, 2003; Tangney et al., 2007; Tracy, Robins, & Tangney, 2007). Furthermore, shame and guilt have been shown to have differential associations with depression (Kim, Thibodeau, & Jorgensen, 2011). Notably, the items in the PERQshame revealed the lowest mean score among the factors. This finding is not unexpected; the sample was a clinical sample in which all of the parents had taken action to undo or repair the consequences of a (their own or someone else's) behaviour by seeking professional help.

The items in the PERQguilt factor seem to be more complex, and the factor includes more than one basic emotion at T1. The items showing the highest loadings onto the factor were the guilt items (hence, the label PERQguilt), but at T1 fear also clustered with the items. One possible reason for this result is that the wording of the fear item (“I have felt afraid since I learned about my child's trauma”) could be ambiguous. Caregivers might, for example, have understood this item to mean either “I feel afraid because I did something wrong” or “I feel afraid because I don't know how to prevent future traumas.” The results concerning the fear item is more equivocal at T2. However, the sample size at this time point is much smaller, something that might limit the results.

### Discriminant validity and the subscales' sensitivity to change

The results of the discriminant validity analyses indicated that the three PERQ scales have satisfactory discriminant validity. Although the analyses supported the unique contribution of the PERQ subscales, several points are worth discussing. First, the strongest correlation emerged between the PERQdistress subscale and the CES-D. This is not surprising given that the wording for several of the items in the distress subscale is similar to items assessing depressive symptoms in the CES-D. Still, we propose that the major difference between the scales is that the PERQdistress measures emotions that are directly related to a trauma experienced by a caregiver's child, whereas the CES-D measures general depressive symptoms. The small correlation between the PSQ subscales and the PERQ subscales, in contrast, indicates that these scales measure conceptually different phenomena.

All three subscales of the PERQ changed significantly from pre- to post-therapy, showing that they were all sensitive to change. However, by investigating the ES of the change, the sum score of the PERQdistress factor showed a considerably larger ES than did the other subscales. Self-reflective emotions (PERQshame and PERQguilt) may be less malleable or may need more time to change than do parents’ distressed and depressive emotional responses to their children's trauma. However, the magnitude of the ES should be interpreted carefully, and there were statistically significant changes (improvements) on all of the subscales from pre- to post-therapy.

### Limitations and further development of the scale

A major limitation of this study was its small sample size. The factor structure of the PERQ needs to be evaluated using confirmatory factor analyses with a larger sample size. Furthermore, the factor loadings for the anger and fear items are equivocal, and findings from replication studies may suggest a revision of the proposed subscales in this study. If relevant for the sample, also item 15 should be included in the analysis. In addition, the time between a given child's trauma and his/her caregiver's screening varied widely. Additionally, although the findings with only the mothers’ data generally supported the factor structure found in the full sample, the small number of fathers compared to mothers in this sample limits the generalizability of the results to fathers. It may be that mothers and fathers respond to their children's traumatic experiences differently. These issues should be addressed in a larger sample. More research is also needed to examine whether the factor structure revealed by the EFA can be replicated in samples of individuals of different ages, traumatic experiences, and cultural backgrounds. Parents may experience different emotional reactions as their children grow older and their roles as protectors change.

Although the PERQ has been a useful tool both in research and clinical work, the scale has potential for further development. The wording of a few of the items may be ambiguous; thus, increased specificity may improve the scale. For example, item 6, “I have felt afraid since I learned about my child's trauma,” could be expanded and divided into the following items: “I have felt afraid that my child will be exposed to a new trauma” and “I have felt afraid that my child will not recover from the trauma.” The guilt items may also be expanded by adding items such as, “I have felt responsible for the negative reactions my child has demonstrated since the traumatic experience.”

## Conclusion and clinical implications

This study identified clinically meaningful PERQ subscales with acceptable psychometric properties. It is important for clinicians to identify, address, and be aware of the distinct emotional reactions represented by the subscales. For example, by identifying parents who feel shame about their children's traumatic experiences, clinicians are in a good position to provide relevant help and to motivate parents who fear the therapist will blame them for their children's trauma to continue the therapy process. Furthermore, although item 15 “I feel guilty that I did not know about the trauma sooner,” was omitted from the analysis in the current study, we consider it as a clinically meaningful item, and recommended that it be used in future clinical practice when applicable.

Although the current study was not able to determine the relationship between parental emotional reactions and their children's experiences, symptoms, and recovery from trauma, clinicians should be attentive to parents’ reactions following traumatic events. In fact, reducing parental distress by normalizing emotional reactions through psychoeducation and by addressing any related cognitive distortions has been demonstrated to correlate with better child outcomes (Cohen et al., 2004). Hence, in addition to supporting parents so that they may become a resource for their child, clinicians should help parents regulate their own emotions. Although these two processes seem separate, they are intertwined and accomplish the same goal, namely, helping the child. To reach the goal of supporting the child through supporting the parent in clinical practice, the PERQ subscales are helpful resources and should be used individually for each parent, knowing that different parents may differ significantly as to how they react and feel when their child has experienced a traumatic event.

## Appendix

The English version of the revised PERQ.

 1. I have felt upset about my child's trauma.

 2. I think about what happened to my child while I am working.

 3. I have felt sad about my child's traumatic experience.

 4. I am afraid of what other people will think about my child's traumatic experience.

 5. I feel that I should have been able to keep the trauma from happening.

 6. I have felt afraid since I learned about my child's trauma.

 7. I have trouble falling asleep at night because I think about what happened to my child.

 8. I have felt angry about my child's traumatic experience.

 9. Since I learned about my child's traumatic experience, I have been having headaches, stomachaches, etc.

10. I have felt embarrassed about my child's traumatic experience.

11. I have cried about my child's traumatic experience.

12. I have felt ashamed about my child's traumatic experience.

13. I have felt responsible for my child experiencing trauma.

14. I have felt insecure since I learned that my child experienced trauma.

15. I feel guilty that I did not know about the trauma sooner.

The Norwegian translation of the revised PERQ.

 1. Jeg har vært oppskaket over det barnet mitt har opplevd.

 2. Jeg tenker på det barnet mitt har opplevd mens jeg jobber.

 3. Jeg har vært lei meg for det barnet mitt har opplevd.

 4. Jeg er redd for hva andre mennesker vil tenke om det barnet mitt har opplevd.

 5. Jeg føler at jeg burde ha klart å hindre det som skjedde.

 6. Jeg har følt meg redd etter det barnet mitt har opplevd.

 7. Jeg har problemer med å sovne om natten fordi jeg tenker på det barnet mitt har opplevd.

 8. Jeg har vært sint for det barnet mitt har opplevd.

 9. Etter det barnet mitt opplevde, har jeg hatt hodepine, vondt i magen, etc.

10. Jeg har vært flau over det barnet mitt har opplevd.

11. Jeg har grått over det som barnet mitt har opplevd.

12. Jeg har følt meg skamfull over det barnet mitt har opplevd.

13. Jeg har følt meg ansvarlig for det barnet mitt har opplevd.

14. Etter det barnet mitt opplevde, har jeg følt meg usikker.

15. Jeg har dårlig samvittighet for at jeg ikke visste om det som skjedde med barnet mitt før.
